# Extinction of an introduced warm-climate alien species, *Xenopus laevis*, by extreme weather events

**DOI:** 10.1007/s10530-015-0944-x

**Published:** 2015-07-16

**Authors:** Richard C. Tinsley, Lucy C. Stott, Mark E. Viney, Barbara K. Mable, Matthew C. Tinsley

**Affiliations:** School of Biological Sciences, Life Sciences Building, University of Bristol, Bristol, BS8 1TQ UK; Institute of Biodiversity, Animal Health and Comparative Medicine, University of Glasgow, Glasgow, G12 8QQ UK; School of Biological and Environmental Sciences, University of Stirling, Stirling, FK9 4LA UK

**Keywords:** Invasive species, *Xenopus laevis*, Extinction, Climate change, Extreme weather

## Abstract

Invasive, non-native species represent a major threat to biodiversity worldwide. The African amphibian *Xenopus laevis* is widely regarded as an invasive species and a threat to local faunas. Populations originating at the Western Cape, South Africa, have been introduced on four continents, mostly in areas with a similar Mediterranean climate. Some introduced populations are also established in cooler environments where persistence for many decades suggests a capacity for long-term adaptation. In these cases, recent climate warming might enhance invasion ability, favouring range expansion, population growth and negative effects on native faunas. In the cool temperate UK, populations have been established for about 50 years in Wales and for an unknown period, probably >20 years, in England (Lincolnshire). Our field studies over 30 and 10 years, respectively, show that in favourable conditions there may be good recruitment, fast individual growth rates and large body size; maximum longevity exceeds 23 years. Nevertheless, areas of distribution remained limited, with numbers <500 in each population. In 2010, only a single individual was captured at each locality and further searching failed to record any others in repeated sampling up to 2014. We conclude that both populations are now extinct. The winters of 2009–2010 and 2010–2011 experienced extreme cold and drought (December 2010 was the coldest in 120 years and the third driest in 100 years). The extinction of *X. laevis* in these areas indicates that even relatively long-established alien species remain vulnerable to rare extreme weather conditions.

## Introduction

Two topics are prominent in the current ecological literature: climate change and invasive species. Recent climate change has been characterised both by increased warming and by increased frequency of ‘extreme events’. The latter are illustrated for England by the coldest and driest winter for 100 years (2010/2011), the highest annual rainfall for 100 years (2012), the coldest spring for 50 years (2013), the wettest winter for over 250 years (2013/2014). Overall, 2014 was the warmest year since records began in 1659 (The Central England Temperature series; http://www.metoffice.gov.uk/climate/uk), and was also the warmest year ever recorded worldwide (data for 1880–2014) (http://www.noaa.gov). These episodes can be predicted to have profound effects on the fauna of affected regions, either releasing environmental checks on life history traits or leading to extinction of species that are vulnerable to perturbation outside narrow environmental boundaries. Invasive non-native species are considered the second biggest threat after habitat loss to biodiversity worldwide; damage caused by invasive species globally amounts to almost 5 % of the world economy (Defra 2008).

These themes of climate change and invasive species may converge in cases of species transfers between different climate zones. Short- and long-term changes in climate parameters may have strong positive or negative effects on the abundance and distribution of translocated species. The literature highlights a wide diversity of species that may become invasive following introduction to new geographical or climate regions (e.g. Davis 2009). Some amphibians have notoriety as invasive species, especially cane toads (*Rhinella marina*) and North American bullfrogs (*Lithobates catesbeianus*) (Stuart et al. 2008). The African clawed frog, *Xenopus laevis*, has been important in biomedical research for over 80 years: this species, native to southern Africa, was employed in human pregnancy diagnosis until the 1960s and is currently a model organism for research (Cannatella and de Sá 1993; Gurdon 1996; Tinsley 2010; Furman et al. 2015). Release or escape into the wild of laboratory animals together with others kept as pets has contributed to establishment of widely-distributed alien populations, now including the USA, Ascension Island, Chile, UK, France, Italy, Portugal and Japan (Tinsley and McCoid 1996; Lobos and Measey 2002; Crayon 2005; Kobayashi and Hasegawa 2005; Lobos and Jaksic 2005; Fouquet and Measey 2006; Faraone et al. 2008; Rebelo et al. 2010; Measey et al. 2012; Lillo et al. 2013; Lobos et al. 2013). These introductions are considered a threat to local faunas (e.g. Lafferty and Page 1997; Lillo et al. 2011; Amaral and Rebelo 2012). Recent concerns focus on the alleged involvement of *X. laevis* in worldwide spread of the fungal pathogen *Batrachochytrium dendrobatidis* implicated in extinction of vulnerable anuran species (Weldon et al. 2004; Fisher and Garner 2007; Goka et al. 2009; Solís et al. 2010; Vredenburg et al. 2013; but see also Tinsley et al. 2015).

In the UK, there have only been two well-established populations of *X. laevis*: in Glamorgan, Wales, and Lincolnshire, England. A third, on the Isle of Wight, was introduced in the 1960s but is now probably extinct (Tinsley et al. 2015). The responses of the relevant government environmental authorities to the extant populations differed in the two regions. In Wales, there was no formal reaction to the presence of a breeding colony, and research published during 25 years did not attract conservation concerns. As a result, there is a substantial body of ecological findings including diet, growth rates, age determination, population biology and parasite infection (Tinsley and McCoid 1996; Measey and Tinsley 1998; Measey 1998, 2001; Tinsley and Jackson 1998; Tinsley et al. 2011a, b, 2012). When *X. laevis* was recorded in Lincolnshire, English Nature (now Natural England) began a programme in 2003 to eliminate the colony. Their approach excluded any allowance for compiling baseline population data: the over-riding priority was for immediate removal. So, ecological studies have been limited. In 2008, the Welsh Government adopted new plans following the Invasive Non-Native Species Framework Strategy for Great Britain (Defra 2008) and an eradication programme began in 2010 in collaboration with the Environment Agency, Wales and the Countryside Council for Wales.

Our fieldwork, beginning in 1981 in Wales and 2001 in Lincolnshire, continued in 2010 with the expectation that the populations in both areas would number several hundred individuals. However, initial observations suggested that both populations might have become almost or completely extinct. To confirm this, intensive searches were undertaken in 2010 and 2011, aiming to survey habitats over a wide area on multiple occasions. This account reports the evidence for the conclusion that *X. laevis* is extinct at both sites and interprets the likely causative factors in relation to exceptional weather conditions, acting in conjunction with specific habitat and population characteristics. The half-century history of this alien species in the UK has wider conservation significance for assessing the ability of warm-climate introduced species to persist during climate fluctuations occurring over decades and, especially, to survive rare extreme events.

## Methods and background

### Fieldwork approach

*Xenopus laevis* were caught with funnel traps baited with raw meat (usually ox liver), partially submerged in water to allow access to air by captured animals (details in Tinsley et al. 2015). Traps were set at around darkness (20:00–22:00 h), inspected at dawn (04:00–05:30 h) and then typically re-set for the following day. Each period when a trap was in situ during the day or night is referred to below as a ‘trap-session’. Typically, on each fieldwork visit, trapping was repeated over several consecutive sessions (up to 6 in succession in the Lincolnshire ditch and Croescwtta pond) with traps set at high density (usually 1.5–2 m apart). In addition, some animals in Lincolnshire were caught in torchlight at night using long-handled pond nets. Numbers of *X. laevis* cited in the text and figures refer to adults and juveniles captured (not projections of population size); tadpole numbers were not recorded. Animals caught in Wales contributed to a mark-recapture programme carried out over 30 years using two methods for individual identification. Initially (1981–1994), unique combinations of spots of alcian blue dye were produced with a Panjet (Wright Health Group Ltd., Dundee, UK); in subsequent years, marks were freeze-branded letters and numbers (see Supplementary Information in Tinsley et al. 2015). Both marking methods depended on the tough skin characteristic of *X. laevis* and the light background provided by the ventral surface. The marks remained clearly visible throughout the period for which individuals were followed, i.e. up to 23 years for the dye marks and 15 years for the freeze-brands, but the combinations of letters and numbers (3 per animal) allowed quicker recognition, especially during night-time fieldwork. Age was determined by skeletochronology for animals in Wales and Lincolnshire and, in Wales only, by records of individuals marked soon after metamorphosis and followed with re-captures in subsequent years (Tinsley et al. 2012, 2015). Procedures were approved by the Ethics Committee at Bristol University and carried out under UK Home Office Licence. Water temperatures (generally at depths of 20–60 cm) were recorded with Tinytag data loggers [Gemini (UK) Ltd].

### Location and environmental conditions

Habitat characteristics are important for understanding the environments in which introduced *X. laevis* persisted in the UK and in which they ultimately went extinct; we provide details of the sites to illustrate prevailing conditions.

#### Wales

**Fig. 1:**
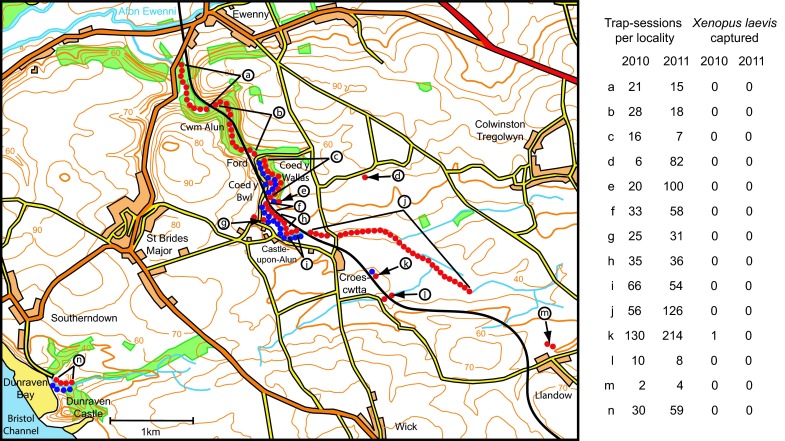
Distribution of *Xenopus laevis* and of fieldwork trapping in Wales: Alun valley and surrounding area, centred on 51°27′50″N, 3°34′14″W, south of Bridgend, Mid-Glamorgan. *Blue circles* sites of occurrence of *X. laevis* in the period 1981–2008 (see text for specific years of records). *Red circles* positions of traps in 2010 and 2011 with numbers of trap-sessions in each locality, *a*–*n*, and numbers of *X. laevis* caught. *Paired lines* group together data for a series of habitats, *single lines with arrows* indicate specific ponds

The fieldwork area centres on the Alun valley, near Bridgend, adjacent to the Bristol Channel (Fig. 1). A plateau of Liassic limestone (40–90 m a.s.l.) supports pasture farmland from which the river Alun descends into a wooded valley in Carboniferous limestone. The drainage experiences extremes of water flow: after prolonged periods without rain the river disappears into underground fissures and the riverbed is dry, but heavy rain produces torrential floods, often 1–2 m deep. The area has stone-built livestock watering ponds constructed in the 19th century, typically over a spring or seepage that maintains permanent water even during drought. One such site is a pond at Croescwtta farm (Fig. 1k), area 110 m^2^, located about 400 m up-slope of the river course but without direct connection to it. In the 1990s, water depth was about 60 cm but sediment accumulated so that, during drought in the late 2000s, the water was only a few centimetres deep above about 1.2 m of anoxic mud. Water temperatures on the pond substrate in 2006–2008 were <10 °C for over 6 months each year (mid-October to late April), >15 °C for 4–5 months and >18 °C for only a few days (Tinsley et al. 2011a). *Xenopus laevis* may experience warmer conditions (but a greater risk of predation) in surface water in sunshine, up to 23 °C (Tinsley et al. 2011b).

Habitats surveyed in 2010 and 2011 (Fig. 1) can be considered in 3 categories: (A) localities with recent occurrence of *X. laevis* (within the preceding 5 years; Fig. 1e, g, k); (B) localities with previous occurrence (principally 1980s/early 1990s) but without recent records (c, f, h, i, j, n); and (C) habitats apparently favourable for *X. laevis* but with no evidence of colonisation (a, b, d, l, m).

Trapping was possible during multiple fieldwork visits at sites with permanent water (i.e. the stone-built ponds at e, g, k; Fig. 1): for example, at Croescwtta, there were 7 trapping sessions over 5 months in 2010. In the Afon Alun, the presence of water in the river course is dependent on rainfall, restricting the opportunities for trapping: fieldwork was timed to exploit favourable conditions for water depth, flow rate and temperature. Optimal conditions in 2010 occurred in May and June in the Alun tributaries (Fig. 1j), in July and August in the middle Alun (c, f, h, i), and in early September from the ford downstream to Ewenny (a, b). Overall, there were 12 fieldwork nights and 478 trap-sessions between mid-April and October 2010. In 2011, most of the Afon Alun was dry from March to early July, and the riverbed remained dry downstream of Coed y Bwl until early September. Trapping was undertaken during 3 periods (5, 5 and 4 nights/visit) following heavy rainfall: 2 in July and 1 in September 2011, total 812 trap-sessions. Two single-night visits, in August 2013 and August 2014, surveyed the stone-built ponds (each with 26 trap-sessions).

#### Lincolnshire

**Fig. 2:**
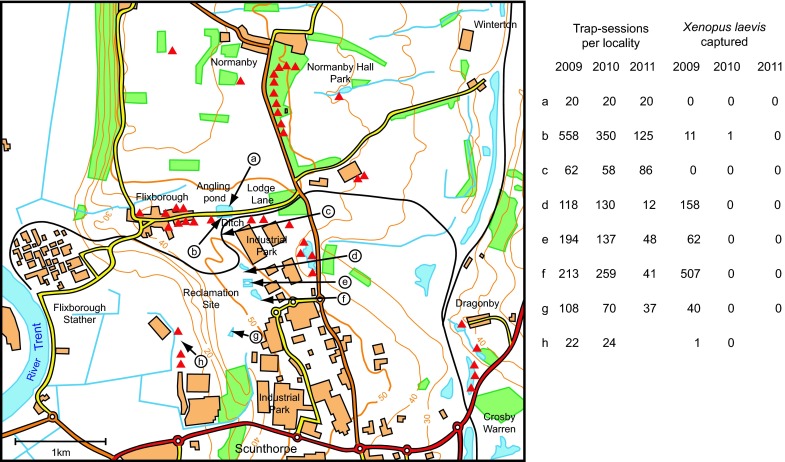
Fieldwork area in North Lincolnshire, centred on 53°37′30″N, 0°40′14″W, north of Scunthorpe. *Red triangles* show localities of trapping in 2003–2010 where *Xenopus laevis* was never recorded (most sites trapped repeatedly in successive years). Data on the right of the map record habitats in which *X. laevis* occurred and results of surveying in 3 years, 2009–2011, showing numbers of trap-sessions at each site (*arrows* to *a*–*h*) and numbers of *X. laevis* caught

The habitats of *X. laevis* lie on an escarpment, mostly around 50 m a.s.l., north of Scunthorpe. The area is divided into two landscape types with an abrupt border along Lodge Lane (Fig. 2). Land use north of this road is agricultural, principally arable farmland and woodland; land to the south was previously dominated by a steelworks but has undergone major re-development as an industrial park. Parts of the steelworks site were heavily contaminated by chemical waste and re-development included removal of toxic soil. During our fieldwork, the ‘reclamation site’ had extensive spoil heaps and temporary ponds but the less-disturbed margins formed wetland habitats with reedbeds. The angling pond and ditch (Fig. 2a, b) had deep permanent water but most ponds on the reclamation site had shallow water, drying out during drought. There was no flowing water at any sites occupied by *X. laevis* and no direct connection with rivers. Water temperatures in the ditch, monitored during 2004–2008, were comparable with those recorded in Wales.

Habitat characteristics at the ditch are significant because survival of *X. laevis* was recorded here when extinction occurred elsewhere across the area. The ditch, 120 m long, is V shaped in section, 2–4 m wide, with one side formed by the vertical wall of a former quarry and the other bank created by in-filling of the quarry to within a few metres of the cliff. Maximum water depth was >3 m (eastern end), becoming gradually shallower towards the west (Flixborough). The site had a rich invertebrate fauna. Fish, potential competitors/predators of *X. laevis*, were absent. There were abundant refuges (in the cliff-face of the original quarry) and dense pondweed provided cover for adults from aerial predators and for tadpoles from cannibalism.

Trapping in 2010 and 2011 focused on habitats in 2 contiguous areas known to have supported *X. laevis*, centred on Lodge Lane (Fig. 2a, b) and the ‘reclamation site’ (d–h), together with a drainage channel (c) providing a potential migration route between these areas but in which there had been no previous captures. Fieldwork was timed to follow rainfall, providing optimum conditions in both years: there was no wind or rain to disturb traps; clear water gave good views of the substrate in torchlight at night (when tadpoles, often elusive by day, are conspicuous). In 2010, fieldwork was focused into 3 periods, each of 5 days, in late June, early July and mid-August, with 1048 trap-sessions. Low rainfall in early summer 2011 led to contraction of waterbodies, potentially concentrating any *X. laevis*; fieldwork after rain in mid-July included 369 trap-sessions over 5 nights.

## Results/observations

### Wales

#### Characteristics of the introduced populations prior to 2010

*Xenopus laevis* was first seen in Cwm Alun in 1966 and a breeding population was well-established in the 1970s (Gillham 1989). Our field studies, in part with Mark Simmonds, John Measey and Joseph Jackson, included a mark-recapture programme involving approximately 2000 individuals followed during the 30 year period, 1981–2010. In the early 1980s, *X. laevis* was found in a stream at Dunraven Castle and along the Afon Alun and associated ponds from the ford at Coed y Wallas upstream to near Croescwtta (Fig. 1). About 250 individuals were marked, almost all adult, at or near full size when first caught. Breeding was observed at several sites but offspring were rarely recorded beyond metamorphosis. In the later 1980s, the number of *X. laevis* caught declined. This coincided with habitat reduction (including in-filling of ponds in disused quarries at Coed y Bwl and Castle-upon-Alun), but the decline also occurred where habitats remained apparently favourable (including Dunraven). During 1990–2000, population decline continued in the areas of original occurrence but a new concentration of *X. laevis* was found in 1994 in an isolated pond at Croescwtta farm (Fig. 1k): mark-recaptures over 2 years recorded >900 individuals, most resulting from spawning in 1993 (Measey 2001). Numbers caught at Croescwtta pond fell to <150 in the early 2000s, but successful recruitment (especially in 2005 and 2006) boosted the population to >400 adults and juveniles by 2008.

#### Expectations of population size in 2010

Predictions of numbers of *X. laevis* likely to be caught were based on the preceding 30 year record, including the following previously unpublished data:

- Total numbers of adults/juveniles individually-marked during the three decades were (approximately): 1980s, 350; 1990s, an additional 1000; 2000s, an additional 500.
- Recapture records in the Afon Alun showed individual longevity exceeding 23 years. At Croescwtta pond, >80 animals first caught and marked in 1994/5 (Measey 2001) were still present in the mid-late 2000s (with ages ≥15 years).
- Year-to-year survival rates are indicated by recaptures of a single cohort of *X. laevis* in Croescwtta pond, born in summer 2005 (total marked 225) and followed until July 2008. For instance, 189 individuals were caught on a single night in September 2006; of these, 171 (90.5 %) were still alive in August 2007 (after 48 weeks) and 163 (86.2 %) in October 2007 (after 56 weeks).
- Population numbers in Croescwtta pond are indicated by sampling for parasitological studies (e.g. Tinsley et al. 2012) based on captures with 14 traps, set for c. 5 h (21:00–02:00 h) on a single night. In August 2007, October 2007 and July 2008, total captures were 340, 345 and 319 respectively.
- With less intensive surveys in the Afon Alun during the mid-2000s, predicted numbers of *X. laevis* in the Afon Alun were more uncertain, but the population decline evident in the later 1980s appeared to have continued. However, factors favouring persistence included, at least in the Alun habitats, long recorded lifespan (b, above) and possible downstream dispersal from the Croescwtta population.

Even if expectations were based principally on Croescwtta, the high survivorship would give confidence that several hundred *X. laevis* should have remained in this area in 2010.

#### Fieldwork outcome in 2010 and 2011, extended in 2013 and 2014

The survey of habitats (listed in Fig. 1), involving 1290 trap-sessions in 2010 and 2011, resulted in a single capture of *X. laevis* at Croescwtta pond on 19 May 2010. This was a male, first recorded and marked in July 2005 when its body length (SVL) was 68 mm, probably metamorphosed in 2003. It was caught 9 times up to its final appearance, including every fieldwork visit from May 2006 to July 2007 (7 sessions in succession), with final SVL 77 mm, body weight 43 g. This equates to ‘large body size’ for a male 7 years old in the Welsh population. At autopsy, it had fat bodies weighing 1.14 g, reflecting excellent nutritional condition. It had no external indications of past pathogenic infection, trauma or unfavourable environmental conditions. Apart from this capture, all other trap-sessions in the 2 years over the entire area, together with the more limited sampling in 2013 and 2014 (52 trap-sessions), were negative.

### Lincolnshire

#### Characteristics of the introduced populations prior to 2010

The timing and source of original introduction are unknown but at both Lodge Lane and the reclamation site large breeding populations were already well-established when fieldwork began. The first record was in 2000 when the Scunthorpe Police contacted R.C.T. to report captures of *X. laevis* on rod and line in a pond managed by the Police Angling Club. Field visits in 2001 and 2002 confirmed that a population occurred here and in an adjacent ditch. The control programme instituted by Natural England began in 2003, both to remove *X. laevis* from known sites and to determine wider distribution. The data below are based on numbers of animals captured and removed during this eradication programme.

Trapping at the fishing pond led to rapid decline of a limited population: 61 individuals removed in 2003, 21 in 2004, then 1–3 per year 2005–2007 and none subsequently. Reports from the Angling Club indicated that no *X. laevis* were caught with rod and line in the pond after 2007. Trapping in the ditch removed 456 adults/sub-adults in 2003 and 421 in 2004 and this largely eliminated recruitment; spawning was halted entirely from 2007 and numbers of adults/sub-adults caught were reduced to 11 in both 2008 and 2009.

Adult *X. laevis* at the ditch were distinguished by very large body size: in every year from 2003 to 2009, females were caught with snout-vent length (SVL) >100 mm and body weight >100 g (maxima 113 mm, 134 g), and males up to 91 mm SVL, weight 65 g. Bone growth ring analysis indicated that large size may be reached in 5–6 years, reflecting fast growth rates.

At the reclamation site, the single season of trapping in 2009 (4 fieldwork visits in early and late July, August and September, total 17 days) removed 768 adult and juvenile *X. laevis* and many hundreds of tadpoles (Fig. 2). Body sizes were smaller than in the Lodge Lane ditch (maximum SVL 85 mm for females, 65 mm for males).

All other potential habitats within a few kilometres of the Lodge Lane population were surveyed during 2003–2009. Trapping was carried out over successive years at most localities, including: ponds near Normanby village, ornamental waterways and a fishing lake in Normanby Hall Country Park, garden ponds in Flixborough village, and effluent ponds associated with poultry farms (Fig. 2). A network of shallow drainage ditches could connect these sites during heavy rainfall, facilitating migration of *X. laevis*. Trapping was also undertaken in more distant localities, including flooded quarries near Dragonby (Fig. 2) and ponds at Burton-upon-Stather and Thealby (about 5 km north and north-east). All these wider searches for *X. laevis* were entirely negative.

#### Expectations of population size in 2010

Predictions of numbers remaining relied on extrapolation from the previous years’ removals. For the angling pond, it was probable that no *X. laevis* would be caught: trapping had been negative since 2007 and immigration appeared to have stopped following depletion of its source in the ditch. However, the population in the ditch had not been eliminated entirely (11 adults in each of 2008 and 2009). So, a small residue was still expected in 2010, with the possibility of resurgence if breeding were successful in an otherwise ‘vacant’ habitat. At the reclamation site, although >700 individuals were removed in 2009 (Fig. 2), numbers caught towards the end of this season still exceeded 50 per fieldwork visit, so some would have remained in 2010. In addition, despite mass captures, large numbers of *X. laevis* tadpoles completed metamorphosis in late summer/early autumn 2009 and their continued survival would have benefited from removal of cannibalistic adults. Overall, a population of several hundred animals would have been expected in 2010.

#### Fieldwork outcome in 2010 and 2011

In total, the 1417 trap-sessions carried out over the 2 years (Fig. 2) resulted in capture of a single *X. laevis* in Lodge Lane ditch on 23 June 2010. There were no other captures in this habitat during intensive trapping in June, July and August 2010 totalling 350 trap-sessions, and no observations of adults or tadpoles during night-time searches. The individual was a juvenile male, with SVL 46 mm and body weight 9.8 g, suggesting metamorphosis in the previous year. Body condition was good, without evidence of pathological effects of disease, malnutrition or severe environmental conditions. All other trapping over the fieldwork area in 2010 and 2011 was negative (Fig. 2). Further confirmation that *X. laevis* was absent from the angling pond was provided by the ‘continuous sampling’ undertaken by anglers: no captures were reported up to late 2014.

### Observations on native species in the same habitats

Studies on native amphibians were incidental to the main remit of trapping *X. laevis* in Wales and Lincolnshire but records were compiled of *Rana temporaria*, *Bufo bufo, Triturus helveticus, Triturus cristatus* and *Lissotriton vulgaris* captured in baited traps and seen on land and in water at night. At both localities, native amphibians appeared abundant, especially on the derelict industrial land in Lincolnshire; records for specific sites are listed by Tinsley et al. (2015). The annual field studies suggested no major changes in frequency of encounters with native species over the final 10 years of fieldwork in both Wales and Lincolnshire. After the severe winters of 2009/2010 and 2010/2011, populations of adult native amphibians were still abundant in the following summers and large tadpole populations indicated successful spawning.

Eels (*Anguilla anguilla*) were relatively common in the Afon Alun during the 1980s, captured in baited traps together with adult *X. laevis* and also seen in torchlight along the riverbed. However, they were not caught during occasional surveys of the Afon Alun in the early 2000s nor during the intensive trapping along the length of the river in 2010 and 2011. Eels were never seen in the Lincolnshire sites.

## Discussion

### Evidence of extinction

Our studies of introduced *X. laevis* extend over 30 years in Wales and 10 years in Lincolnshire. Both populations were undoubtedly established for considerably longer: probably for about half a century in Wales. They would both be judged successful in specific habitats in terms of recruitment, long-term survival and fast body growth rates despite a temperature regime that is cool for a species native to southern Africa (Tinsley et al. 1996). At the end of the first decade of the 2000s, it could be predicted that populations of several hundred adults would occur in both areas, including individuals resident at the same site (in Wales) for over 15 years. A single *X. laevis* was caught in the initial fieldwork in each of the areas in 2010; after this, there were no further captures. In addition, there were no sightings of the distinctive tadpoles which are conspicuous in open water and would have indicated presence of the more elusive adults. Disappearance followed exceptionally severe winter conditions, with temperatures and rainfall amongst the lowest recorded in England and Wales. This event demonstrates that even apparently well-established introduced species may remain vulnerable to rare natural perturbations in environmental conditions.

The ecological characteristics of the UK habitats conform with the general features influencing the distribution of *Xenopus* species in Africa and in areas of introduction elsewhere in the world (Tinsley and McCoid 1996). *Xenopus* species are rarely successful in habitats occupied by large fish populations but they thrive in disturbed landscapes and in man-made habitats: domestic and agricultural water sources (wells, irrigation dams) and ponds created by surface excavation. Typically, the temporary habitats lack established populations of competitors and predators (Tinsley et al. 1996).

In Lincolnshire, some adult *X. laevis* were initially caught in an angling pond, but breeding was never recorded and these migrants were probably an ‘over-spill’ from the adjacent ditch. Other sites were temporary ponds on derelict industrial land. In Wales, population ecology in the Afon Alun was influenced by intermittent water flow. During drought, *X. laevis* could survive in isolated pools in the river course, in subterranean water bodies and by burying themselves in mud. The river characteristics serve to restrict fish populations with the exception of eels that, exactly like *Xenopus* spp., can migrate overland, tolerate drought, and exploit a scavenger diet (Tinsley et al. 1996). Although the population of *X. laevis* in the Alun river was well-established at the start of studies in 1981, it declined steadily and few were caught in the river itself in the 1990s. All the long-term records of populations that recruited successfully were based in artificial pond habitats.

Despite the apparent adaptability of *X. laevis* in the two contrasting landscapes in Wales and Lincolnshire, there are also characteristics of population ecology that emphasise the vulnerability of this species in the UK. First, *X. laevis* has limited migration ability under typical environmental conditions in both localities. In Wales, most individuals marked and recaptured in the final 15 years of monitoring occurred in a single pond (ultimately over 400 animals in an area of only 110 m^2^, see Tinsley et al. 2012). This might have been expected to be a source of emigration but comprehensive surveying suggested little dispersal: Measey and Tinsley (1998) recorded only 2 individuals marked at Croescwtta and found in the Afon Alun in the mid 1990s. In 1999–2010, there were 2386 captures/recaptures of marked individuals in the pond but no recaptures elsewhere (Tinsley et al. 2012 and this account). This parallels the records in Lincolnshire where no *X. laevis* were found in ponds in arable farmland, woodland or gardens adjacent to the industrial sites, all within the potential overland migration range recorded in Africa and California (Tinsley and McCoid 1996).

Second, successful recruitment occurs irregularly. Although *X. laevis* may spawn in most summers, Measey and Tinsley (1998) estimated that new cohorts had established in the Alun valley in only 4 out of 20 years: high tadpole mortality may be attributed to cannibalism and to habitat drying. However, in the later years of the Welsh study, there were major increments to population numbers from successful metamorphosis and high juvenile survival, probably influenced by a succession of warm summers. Nevertheless, even where recruitment may be intermittent, persistence is favoured by long lifespan (over 20 years in Cwm Alun) and occasional large reproductive output. Against this background, the disappearance of both populations was unexpected.

The use of baited traps for live capture of *X. laevis* is highly effective (e.g. Tinsley et al. 2012). The approach is selective: native amphibians found occasionally in traps, can be released without harm; there are no wider biocidal effects (as with use of toxic chemicals), and no disruption of fragile habitats (as occurs with nets dragged over the substrate or through aquatic vegetation). *Xenopus* species can locate prey by odour detection (Tinsley 2010) and the speed of attraction to bait is indicated by captures in Wales where >300 *X. laevis* were caught during trap-sessions of only 5 h. Traps were typically set at high density on several consecutive nights. These repeated sessions should exclude the possibility that animals were missed (for instance, because of reduced attraction to bait during spawning or after a previous natural meal). In both localities, the area surveyed by trapping was larger than the known distribution of *X. laevis* and allowed for flood-borne dispersal, migration along water courses and overland movements to isolated habitats (Figs. 1, 2).

The trapping effort benefitted from optimum fieldwork conditions in both 2010 and 2011. Water temperatures were relatively high, which should have facilitated active foraging by *X. laevis.* In both areas, spring and summer drought would have concentrated *X. laevis* populations but fieldwork was timed to follow the return of water after rainfall. Animals previously confined in habitats with progressively reducing prey would have been readily attracted to baited traps. Based on 30 years’ experience of fieldwork on *X. laevis* in UK habitats, the trapping intensity, duplicated over the 2 years of this search, was far above that required to detect surviving animals: overall totals of 1290 trap-sessions in Wales and 1417 trap-sessions in Lincolnshire. The follow-up surveys confirmed continuing absence up to 2014, both in Wales (focusing on the permanent ponds most likely to provide refuges for any surviving *X. laevis*) and in Lincolnshire (the lack of catches of *X. laevis* by anglers at the fishing pond). Thus, these data provide conclusive evidence of extinction in the areas surveyed.

### Causes of extinction

The disappearance of *X. laevis* from the Alun valley may be interpreted as the endpoint of a long decline. Largest numbers over our 30 year study period were recorded at the start and then fell continuously. The river represents a poor habitat: frequent droughts restricted the periods of feeding, growth and storage of reserves and prevented recruitment. Habitat modification, especially in-filling of ponds, eliminated sites that supported breeding in the early 1980s. As the population declined, extant habitats previously occupied by *X. laevis* were not re-colonised in the valley. However, population characteristics in the pond at Croescwtta were different: over 1000 individuals were monitored here from 1994 (Measey and Tinsley 1998; Measey 2001; this account) and a subset of this population was present throughout the record, with ages >15 years at the end (Tinsley et al. 2012). Mark-recapture data showed high year-to-year survival and numbers were maintained by successful recruitment without evidence of significant emigration. Disappearance of *X. laevis* from this site would have required an exceptional perturbation capable of eliminating all but a single individual over a short period.

The long-term records of individually-marked animals in Wales (e.g. Tinsley et al. 2012) give no indication of an intrinsic factor with the potential for causing rapid, almost total, mortality. No highly pathogenic infections were recorded. Mortality factors affecting *Xenopus* in natural and introduced populations include cannibalism and predation. In the UK, *X. laevis* may be eaten by typical fish and amphibian predators including herons, introduced mink (*Mustela vison*) and, possibly, grass snakes (*Natrixnatrix*). Eels are potential competitors and predators of *X. laevis* (see Tinsley et al. 1996) but, although frequent in the Afon Alun in the 1980s, they were never seen in the later years of this study. It is possible that the eel population was reduced by mink and that this invasive predator also contributed to the decline of *X. laevis* in the river. According to local naturalists, mink have been responsible for a major reduction in nesting wildfowl in Cwm Alun. However, the high survival rates of *X. laevis* in Croescwtta pond exclude a significant impact of mink predation during our long-term studies unless mink located this isolated pond during late 2009/early 2010 and remained until almost all *X. laevis* were eaten. Both mink and eels are absent from the Lincolnshire localities so they could not have played a role in that local extinction.

The habitats occupied by *X. laevis* in Lincolnshire have varied characteristics influencing population survival. The Lodge Lane ditch provides highly favourable conditions but ‘predation' exerted by the Natural England eradication programme was intense: trap captures in 2008 and 2009 were reduced to <2 % of 2003–2004 numbers. The ecology of *X. laevis* on the reclamation site was more typical of that in disturbed habitats in Africa and California (Tinsley and McCoid 1996). Spawning was successful but survival was probably poor in shallow ponds vulnerable to drying, predation (especially by herons) and habitat destruction (by bulldozing). The body size range was smaller than in the ditch, probably reflecting poorer nutrient supply and shorter life span. Nevertheless, a population of several hundred adults and juveniles would still have been expected in 2010 and speculation implicating predator-induced mortality would have to account for total removal of *X. laevis* (except for 1 individual) from about 7 sites over several km^2^ over one winter.

The simultaneous disappearance in Wales and Lincolnshire (over 300 km apart) suggests that mortality factors operating in both areas over the same short timescale are more likely than local influences. The winter of 2009/2010 was exceptionally severe. In England and Wales, temperatures were the lowest for 30 years with the mean for January 3 °C below the 1971–2000 mean; there was also low rainfall (60–70 % of average) and prolonged freezing temperatures (http://www.metoffice.gov.uk/climate/uk/). The following winter was even more extreme: across the UK, mean temperature was 5 °C below the 1971–2000 average. In the ‘Central England Temperature Series’, this was the coldest December for 120 years, with the number of days with air frost the highest for over 50 years. December was also the 3rd driest in 100 years with less than one-third of average rainfall in the Midlands and Wales (http://www.metoffice.gov.uk/climate/uk/). If this combination of low temperatures and low water levels was lethal in 2009/2010, then effects would have been exacerbated in 2010/2011. So, even if some individuals survived at the start of 2010 and were not located in the trapping programme, they may not have survived the conditions at the end of 2010.

The survival of *X. laevis* overwintering in ice-covered ponds has been documented in the USA and Europe (Tinsley and McCoid 1996). The ponds in both Wales and Lincolnshire may have surface ice in most winters but the high year-to-year survival rates recorded in Wales (see above) indicate that winter conditions in the UK are not normally lethal. However, the two very severe winters had unusually low rainfall preceding the period of extreme cold. As a result, water levels were very low and freezing temperatures penetrated deep into wetland habitats. *Xenopus laevis* is able to burrow in mud and could normally escape freezing but mortality may have been caused by a combination of low temperature and anoxia. For example, *Rana muscosa* occurs in the Sierra Nevada, California, at altitudes up to 3700 m a.s.l. (Bradford 1983). At the highest elevations, these frogs ‘overwinter’ for up to 9 months beneath ice but mass mortality may occur in lakes where ice cover is exceptionally thick, making the water effectively shallower. ‘Winterkill’ has been attributed to oxygen depletion: respiration during dormancy is entirely cutaneous and, over prolonged periods, frogs may become surrounded by anoxic mud (Bradford 1983).

It remains an anomaly that a single *X. laevis* did survive the 2009/2010 winter in each area. Both individuals were in good physical condition and showed no evidence of past pathogenic infection nor environmental stressors including starvation. Survival of *X. laevis* in the Lodge Lane ditch may be related to habitat characteristics. This and the adjacent angling pond are the only sites where animals could over-winter in deep water. All other sites, on derelict industrial land, are shallow, vulnerable to drying and to penetration of ice into the substrate. One bank of the ditch, originally the upper part of a vertical quarry cliff-face, has crevices providing underwater refuges. Deep water (>3 m) contributed to a buffered environment below surface ice even in severe winter conditions. The presence of only one *X. laevis* in this favourable habitat may be a consequence of the year-on-year depletion over the previous 7 years.

In Wales, Croescwtta pond would previously have provided a deep-water refuge but habitat changes over at least the past 15 years led to progressive accumulation of mud and decrease in water depth. By the late 2000s, water was only a few centimetres deep during drought and ice could have penetrated into the anoxic sediment. Survival of a single individual here may be attributed to ‘chance’—occurrence in a pocket of water or substrate with continuing oxygen availability, where complete immobilisation did not occur.

### Factors influencing distribution and abundance

*Xenopus laevis* occurs naturally in the Mediterranean climate zone in the southwest of the Western Cape Province, South Africa and all, or most, of the introductions to other world regions have probably been from this source (Tinsley 2010; Lillo et al. 2013). Greatest concerns about the invasive species characteristics of introduced *X. laevis* relate to areas with equivalent environmental conditions (including California, Chile, Italy and Portugal). In the UK, *X. laevis* encountered a temperature regime that greatly restricts the annual period of activity. Water temperatures in the Croescwtta pond may remain below 10 °C for >6 months each year (and below 15 °C for 8 months; Tinsley et al. 2011a). Thus, *X. laevis* may have persisted in the UK at the edge of its environmental—especially climatic—tolerance [corresponding with the modelling of suitable climate space by Measey et al. (2012) and the bioclimatic niche models of Lobos et al. (2013)].

The present field data suggest an apparent paradox. In specific habitats in the UK (as at Croescwtta, Wales, and the Lodge Lane ditch, Lincolnshire), *X. laevis* exhibited fast individual growth rates, large body size, good recruitment and high survival. By contrast, the overall populations remained confined, without significant range extension during 30 years of our studies in Wales and 10 years in Lincolnshire. Indeed, the maximum geographical distribution in Wales occurred at—or probably before—the start of our mark-recapture programme and contracted continuously thereafter. In both Wales (see Tinsley and McCoid 1996) and Lincolnshire, small-scale migration was recorded (e.g. between the Lodge Lane ditch and the angling pond), but there was no evidence of dispersal into apparently favourable ponds connected by drainage channels in adjacent farmland (Fig. 2). The low temperature regime may have specific effects on dispersal behaviour. If dispersal into a wider range of habitats had occurred at both sites, especially those with deep water, the present extinction might not have occurred. This failure to expand in distribution and population numbers represents a major contrast with the ecology of introduced *X. laevis* in other regions.

This assessment of the limitations of *X. laevis* in Wales and Lincolnshire parallels fieldwork findings for a third population, established on the Isle of Wight following release in 1962 (reviewed by Tinsley and McCoid 1996). Studies by Mark Simmonds in the early 1980s indicated a population of <100 adults occupying coastal ponds; numbers declined in the mid 1980s and surveys by R.C.T. and M.C.T. failed to locate any *X. laevis* in these habitats in 1988–1991 (unpublished). Although an intensive search equivalent to present fieldwork in Wales and Lincolnshire was not undertaken, it seemed likely that the population had disappeared from the original sites by the late 1980s (Tinsley and McCoid 1996). Large breeding populations of all three native species of newts co-occurred in these ponds and remained abundant after the demise of *X. laevis*: these may have had a negative effect on the breeding success (especially tadpole survival) of *X. laevis.*

McCoid et al. (1993) reported an apparent decline in introduced *X. laevis* in parts of San Diego County, California, that previously supported dense populations. However, periodic fieldwork by R.C.T. and M.C.T. in the following 10 years (unpublished) consistently recorded *X. laevis* in this area, including the specific sites studied by McCoid et al. Invasive populations may fluctuate in regions where environmental conditions are typically favourable, especially in response to habitat modification and short-term drought, but there are no other records that introduced populations of *X. laevis*, once established for many years, may have disappeared.

It is ironic that the outcome intended by the two programmes to eradicate *X. laevis* in the UK has now occurred through natural causes. The extinction of *X. laevis* at its two known localities emphasises that introduced species may remain vulnerable to rare environmental perturbations, especially when a species that has persisted through 50 years of variable conditions is subjected to once-in-100 years extremes. It may be predicted that other introduced species may also have gone extinct during the same winters of exceptional cold and drought.
